# Targeting extracellular and juxtamembrane *FGFR2* mutations in chemotherapy-refractory cholangiocarcinoma

**DOI:** 10.1038/s41698-021-00220-0

**Published:** 2021-09-03

**Authors:** Michael Bitzer, Stephan Spahn, Sepideh Babaei, Marius Horger, Stephan Singer, Klaus Schulze-Osthoff, Pavlos Missios, Sergios Gatidis, Dominik Nann, Sven Mattern, Veit Scheble, Konstantin Nikolaou, Sorin Armeanu-Ebinger, Martin Schulze, Christopher Schroeder, Saskia Biskup, Janina Beha, Manfred Claassen, Kristina Ruhm, Antti Poso, Nisar P. Malek

**Affiliations:** 1 Department of Internal Medicine I, Eberhard-Karls University, Tübingen, Germany; 2 Center for Personalized Medicine, Eberhard-Karls University, Tübingen, Germany; 3 Cluster of Excellence, Image Guided and Functionally Instructed Tumor Therapies, Eberhard-Karls University, Tübingen, Germany; 4 German Cancer Consortium (DKTK) and German Cancer Research Center (DKFZ), Heidelberg, Germany; 5 Department of Diagnostic and Interventional Radiology, Eberhard-Karls University, Tübingen, Germany; 6 Institute of Pathology and Neuropathology, Eberhard-Karls University, Tübingen, Germany; 7 Department of Molecular Medicine, Interfaculty Institute for Biochemistry, Eberhard-Karls University, Tübingen, Germany; 8 Institute of Medical Genetics and Applied Genomics, Eberhard-Karls University, Tübingen, Germany; 9 CeGaT GmbH and Praxis für Humangenetik, Tübingen, Germany; 10 School of Pharmacy, Faculty of Health Sciences, University of Eastern Finland, Kuopio, Finland

**Keywords:** Bile duct cancer, Molecular medicine, Targeted therapies

## Abstract

Intrahepatic cholangiocarcinoma (iCCA) has emerged as a promising candidate for precision medicine, especially in the case of activating *FGFR2* gene fusions. In addition to fusions, a considerable fraction of iCCA patients reveals FGFR2 mutations, which might lead to uncontrolled activation of the FGFR2 pathway but are mostly of unknown functional significance. A current challenge for molecular tumor boards (MTB) is to predict the functional consequences of such FGFR2 alterations to guide potential treatment decisions. We report two iCCA patients with extracellular and juxtamembrane *FGFR2* mutations. After in silico investigation of the alterations and identification of activated FGFR2 downstream targets in tumor specimens by immunohistochemistry and transcriptome analysis, the MTB recommended treatment with an FGFR-inhibiting tyrosine kinase inhibitor. Both patients developed a rapidly detectable and prolonged partial response to treatment. These two cases suggest an approach to characterize further detected *FGFR2* mutations in iCCA to enable patients´ selection for a successful application of the *FGFR* -inhibiting drugs.

## Introduction

Intrahepatic cholangiocarcinoma (iCCA) has emerged as a promising candidate for precision medicine^1,2^. Alterations in the fibroblast growth factor receptor (FGFR) pathway have been identified as a common event in iCCA^3–8^, including *FGFR2* fusion genes in up to 16% of these tumors^1,6,9–12^. Active FGFR2 fusion proteins share a moiety retaining an intact kinase domain and an extracellular dimerization or oligomerization domain at the C-terminus, provided by different fusion partners^13^. Pemigatinib has been the first FDA-approved FGFR-inhibiting drug for the treatment of *FGFR2* fusion-harboring cholangiocarcinoma^14^, followed by infigratinib, recently. However, uncontrolled activation of FGFR2 can also occur by activating mutations that can lead to receptor dimerization and constitutive kinase activation^15^. Yet, identifying activating *FGFR2* mutations to guide treatment decisions has not been in focus outside clinical studies so far. Consequently, these mutations might often be overlooked, withholding patients from potentially beneficial therapies.

Here, we report two patients with advanced iCCA, one harboring an extracellular and one a juxtamembrane *FGFR2* mutation that were identified during the presentation at our academic molecular tumor board (MTB). After in silico investigation of these mutations and the identification of activated FGFR2-downstream targets in the tumor specimens, the MTB recommended treatment with an FGFR-inhibiting tyrosine kinase inhibitor. Both patients developed a rapidly detectable and prolonged partial response to this treatment.

## Results

### Clinical Case 1, extracellular *FGFR2* mutation

**Fig. 1:**
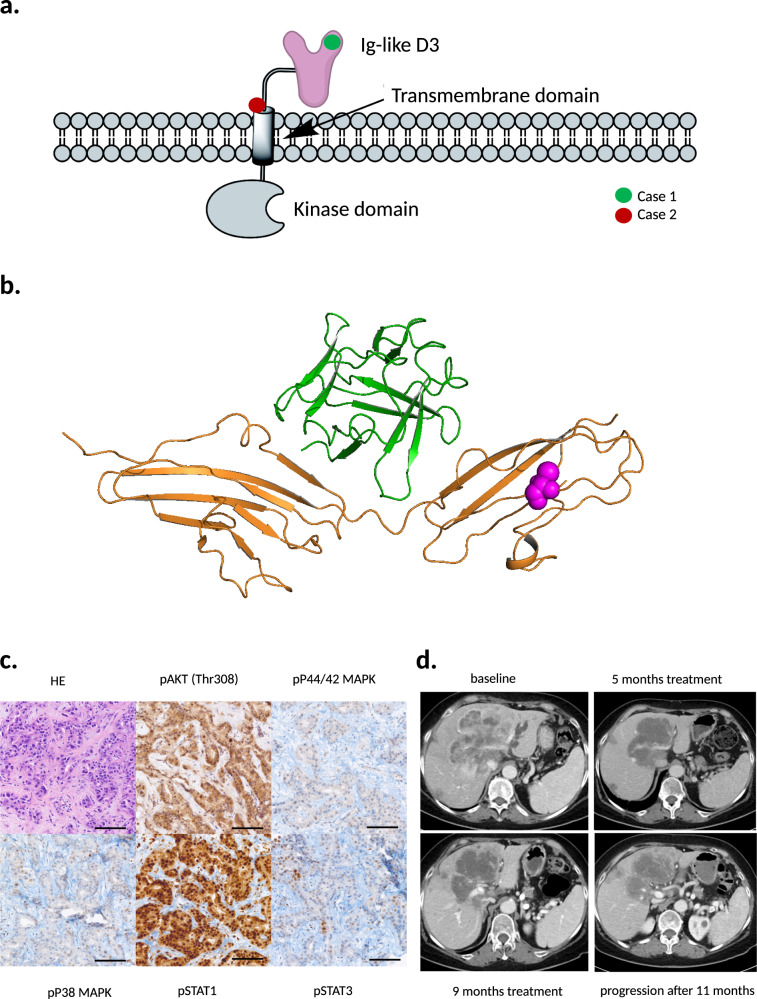
Extracellular FGFR2 alterations, downstream FGFR2 activation in the tumor of Patient Case 1, and clinical course during treatment with pazopanib. **a** Schematic overview of FGFR2 alterations of Case 1 and Case 2 using ChemDraw 19.1. The variant F276C (Case 1) is located within the Ig-like domain 3 of the extracellular receptor region (green circle). The c.1107_1113delinsCTCG alteration (Case 2) is located within the juxtamembrane domain of the receptor (red circle). **b** Structure of FGFR2 with the extracellular variant F276C of Case 1 using Schrödinger Suite 2020-1 and PyMOL Molecular Graphics System, Version 2.0 Schrödinger, LLC. The ligand FGF1 (green ribbon) is bound to FGFR2 (orange ribbon). F276C mutation is shown in the magenta sphere. The mutated residue is located far away from the receptor–ligand interface. **c** Immunohistochemistry of FGFR2 downstream targets in tumor biopsy of Case 1 prior to the treatment with pazopanib. Scale bar 100 µm, magnification x200, objective x20. **d** Before treatment of Case 1 with pazopanib (baseline), a large tumor mass was seen centrally in the liver compressing the left bile duct with secondary upstream bile stasis in a CT scan. The mass showed a slightly heterogeneous enhancement with predominantly hyper-enhancement in the late arterial enhancement phase. 5 months after initiation of pazopanib treatment, the mass showed a significant (>20%) reduction in size and almost complete reduction in tumor enhancement (>80%) and the biliary stasis had vanished. These findings stayed unchanged also 9 months after treatment onset. Progression according to RECIST 1.1 was seen 11 months after treatment initiation.

A 67-year-old female patient was diagnosed with iCCA in April 2018. Shortly thereafter, first-line treatment with gemcitabine and cisplatin (GemCis) was initiated in May 2018. Progressive disease (PD) was found at the first response monitoring with a progression-free survival (PFS) of 2.1 months. Second-line treatment with FOLFIRI was started; however, the first-response monitoring again showed PD with a PFS of 2.4 months. Tumor and normal tissue samples were analyzed by next-generation sequencing of 711 full-coding gene sequences and discussed at the MTB of Tübingen University Hospital, which has been previously described^16^. The sequencing results are shown in Supplementary Fig. 1. No germline alterations were detected. Based on the detection of the extracellular FGFR2 mutation F276C with a high novel allele frequency (NAF) of 0.49 (Supplementary Table. 1), the MTB decided to further investigate FGFR downstream targets by immunohistochemistry (IHC) and an in silico investigation for potential FGFR targeting. The mutation was located in the third Ig-like domain of the FGFR2 extracellular part in a region not directly involved in receptor–ligand interaction (Fig. 1a, b). IHC showed strong phosphorylation and activation of AKT and STAT1, and moderate activation of p44/42 MAPK, p38 MAPK, and STAT3 (Fig. 1c). Based on these results, the MTB recommended an FGFR-directed treatment. Due to a lack of appropriate study options and approved drugs at that time, treatment was started with the FGFR-inhibiting multi-tyrosine kinase inhibitor pazopanib 800 mg once daily based on the data of Borad et al.^7^, which led to a partial response (Fig. 1d). Pazopanib was continued without dose reduction until disease progression, which occurred after 11.0 months.

The Von Hoff model uses patients as their own control, comparing the PFS on a targeted therapy (PFS2) versus the duration of PFS on the last previous therapy (PFS1)^17^. A ratio of PFS2/PFS1 ≥ 1.3 or 1.5 is generally regarded as a clinical benefit^18,19^. PFS2/PFS1 in this patient was highly positive (4.6).

### Clinical Case 2, juxtamembrane *FGFR2* alteration

**Fig. 2:**
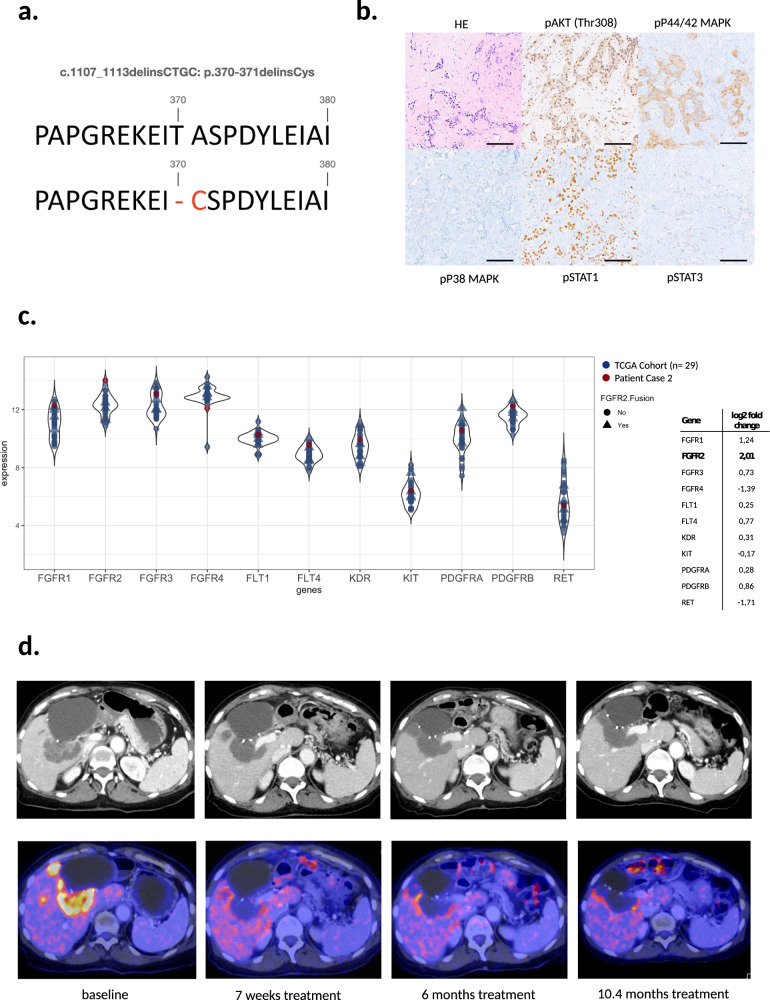
Juxtamembrane FGFR2 in-frame deletion and mutation of Patient Case 2, downstream FGFR2 activation, and clinical course during treatment with lenvatinib. **a** Amino acid change in the juxtamembrane part of FGFR2 caused by the c.1107_1113delinsCTGC; p.370_371delinsCys (ENST00000358487) alteration of Case 2. Upper part: normal FGFR2 protein sequence, lower part: altered sequence. **b** Immunohistochemistry of FGFR2 downstream targets in tumor biopsy of Case 2 prior to the treatment with lenvatinib. Scale bar 100 µm, magnification x200, objective x20. **c** Transcriptome analysis with expression levels of selected tyrosine kinases. Expression levels of the tyrosine kinases FGFR1-4, FLT1, FLT4, KDR, KIT, PDGFRA, PDGFRB, and RET from the transcriptome data of Case 2 (red) and of 29 cholangiocarcinoma patients from the TCGA database (blue). Samples with *FGFR2* fusion genes from the TCGA cohort are shown as a triangle. The table shows the log2-fold change of the patient´s expression level in comparison to the TCGA cohort. **d** Before treatment with lenvatinib, a local relapse of the tumor at the edge of the liver resection area is seen dorsally to a known post-surgical biloma with a partial enhancement of the mass in a CT scan. The lesion showed a late arterial enhancement phase by a predominantly heterogeneous tumor attenuation (baseline, upper row). An additional ^18^F-FDG-PET examination was performed with a highly positive PET signal (lower row). After 7 weeks of treatment with lenvatinib the mass decreased, while the contrast medium enhancement (upper row) and the FDG uptake (lower row) totally disappeared. Note, a second mass next to the local relapse showed identical behavior. After 6 months of treatment, the two tumor areas coalesced, whereas they stayed unchanged in terms of water-equivalent attenuation (lack of solid, enhancing tumor parts) and loss of FDG-uptake. The last scan after more than 10 months showed an ongoing response to treatment.

A 72-year-old female patient was diagnosed with iCCA in April 2018. She received a left-sided liver resection followed by an adjuvant treatment with GemCis over 4 months. New intrahepatic lesions were detected 4 months later and FOLFIRI was started. Unfortunately, progression was already seen at the first follow-up scan, leading to a PFS of 3.0 months. After a biopsy was taken for NGS panel sequencing, she was reexposed to GemCis. Again, progression was detected at the first follow-up scan with a PFS of 2.4 months. In the meantime, sequencing results (709 gene panel) were discussed at the MTB. The sequencing results are shown in Supplementary Fig. 2. A c.1107_1113delinsCTCG alteration of *FGFR2* was identified, leading to a p.370/371 one amino acid in-frame deletion and Cys substitution (Fig. 2a and Supplementary Tab 2). IHC validation on tumor samples showed an activation of the FGFR2 downstream targets AKT, p44/p42 MAPK, p38 MAPK, STAT1, and STAT3 (Fig. 2b). The in silico investigation revealed a location of the alteration at the extracellular juxtamembrane domain (Fig. 1a), with no hint of influencing the binding of FGFR2-targeting tyrosine kinase inhibitors (TKI). To get an impression on the activation level of FGFR2 and downstream targets, transcriptome analysis of a tumor tissue sample was compared to 29 available transcriptome datasets from patients with iCCA of the TCGA cohort (Supplementary Table. 3 and Supplementary Fig. 1a). The PCA plot of this analysis showed that the patient was not an outlier compared to the TCGA samples (Supplementary Fig. 1b). Of note, FGFR2 expression was highest in the patient’s tumor than in all other tumors from the TCGA cohort (log2 fold change = 2.01), whereas the expression levels of different tyrosine kinases, including FGFR1, 3, and 4, was in the range of the other samples (Fig. 2c and Supplementary Fig. 1c). Additionally, the essential FGFR2 downstream targets FRS2, PLCG1, and PIK3CA also showed the highest expression level than all other samples from the TCGA cohort (Supplementary Fig 1c). These data suggest an apparent activation of the FGFR2 pathway in this tumor.

Based on the molecular results and the additional information from IHC, in silico investigation, and transcriptome analysis, the MTB recommended therapy with an FGFR targeting tyrosine kinase inhibitor. Due to the lack of approved drugs and missing study options for this patient, Lenvatinib was chosen as *an off-label* treatment based on its activity against FGFR1-4 and well-reported tolerability in patients with another kind of primary liver cancer^20,21^. With a body weight of 50 kg and a height of 156 cm, Lenvatinib was started with 8 mg once daily. Higher dosages were not possible due to continuous diarrhea at a dose of 12 mg. The first follow-up FDG-PET CT scan showed a significant morphologic and metabolic response to therapy (Fig. 2d), which was still ongoing after 11.6 months. At baseline, the ^18^F-FDG-PET examination showed a highly positive PET signal indicating a significant tumor burden. After 7 weeks of treatment with lenvatinib the mass had shrunken (about 10%), while the contrast medium enhancement (Fig. 2d, upper row) and the FDG-uptake (Fig. 2d, lower row) completely disappeared. The PFS2/PFS1 ratio with >4.8 documents the clinical benefit in this second case.

## Discussion

FGFR receptors consist of an extracellular ligand-binding region with three Ig-like domains, and an intracellular tyrosine kinase domain. Ligand binding induces dimerization and autophosphorylation of the receptor and the subsequent activation of phospholipase Cγ, STAT, PI3K-AKT, and RAS-MAPK signaling.^15^ Small molecules that inhibit the receptor kinase can be divided into non-selective multi-targeted or FGFR-selective TKIs^8,22,23^. First-generation compounds with FGFR-inhibitory activity are non-selective TKIs (e.g. ponatinib, brivanib, nintedanib, dovitinib, lucitanib, pazopanib, or lenvatinib) that inhibit also other tyrosine kinases, including VEGFRs, PDGFRs, FLT3, RET, KIT, or BCR–ABL^24^. Several FGFR-selective TKIs are currently under clinical investigation in iCCA, such as pemigatinib^25^, infigratinib (BGJ398)^26^, erdafitinib^27^, derazantinib^28^, or TAS-120^29^, with pemigatinib being the first drug that received approval by the FDA for CCA with *FGFR2* fusion genes^14^.

FGFR signaling can be aberrantly activated in tumor cells by fusions, missense mutations, or other alterations in genes encoding FGFR family members^15^. Several investigations identified a subgroup of iCCA tumors, mainly with *FGFR2* fusions, that are addicted to FGFR2 activation^15,25,26^. In a recent phase-II study, 36% of the patients with *FGFR2* fusions or rearrangement achieved objective responses to pemigatinib. This study led to the approval of pemigatinib for the treatment of CCA with an *FGFR2* fusion or other rearrangement^14^. In previous studies, nearly all CCA patients who responded to FGFR-inhibiting drugs had tumors with *FGFR* fusion genes. The limited number of reports exploring therapeutic FGFR inhibition in patients with *FGFR* amplification or mutations have been disappointing so far^15,25,26^. In a phase-II study with the FGFR-specific TKI infigratinib, out of 59 patients with *FGFR2* alterations, eight patients had *FGFR2* mutations. Among them, only one patient showed a reduction in tumor size upon infigratinib treatment but did not reach an objective response. This study, however, did not functionally characterize mutations, e.g. by analyzing gene expression or activation of downstream targets^26^.

We present here two cases with different FGFR2-activating mutations that responded to an FGFR-inhibiting TKI. In silico investigations of both mutations did not reveal a hint for a relevant interference of TKI binding to the kinase domain. IHC results showed clear signs of activation for AKT, STAT, and MAPK pathways in both patients. Additional investigations also detected a nuclear accentuated YAP1 and a membrane-associated FRS2 staining for both tumors (Supplementary Fig. 2). Moreover, transcriptome analysis of the second patient and comparison to a TCGA cohort demonstrated a high expression of FGFR2 and of downstream targets such as FRS2, PLCG1, and PIK3CA. Thus, both patients revealed evidence for a constitutive activation of the FGFR pathway.

It was previously reported that a CCA patient with an F276C mutation revealed a partial response to infigratinib lasting around 6 months^30^. Although consequences of this activating mutation were investigated in cellular models, no characterization of patient-derived tumor samples was performed. Furthermore, to the best of our knowledge, the mutation in the FGFR2 juxtamembrane region of patient 2 has not yet been reported in any tumor, nor have FGFR downstream activation and response to FGFR inhibition been analyzed so far. Due to a lack of available specific *FGFR* inhibitors, the MTB recommended treatment of our patients with nonselective TKIs with a high affinity to FGFR2. It cannot be ruled out that the therapeutic effects seen in these two patients are partly supported by inhibition of additional tyrosine kinases. However, both patients had a rapid tumor progression under two prior lines of chemotherapies but reached a substantial partial response with an increased PFS2/PFS1 ratio as an intraindividual sign of therapy response after initiation of the MTB-recommended treatment. Thus, we demonstrate that both mutations are therapeutically targetable by TKIs. It is noteworthy that our two patients should not have been given the drug under normal circumstances, since delins or missense mutations are not yet considered in the FDA label of pemigatinib for CCA treatment.

Intriguingly, mutations of both patients are gain-of-cysteine mutations. The extracellular Ig-like domain 3 of FGFR2 contains a disulfide bond between C278 and C342. It is conceivable that the F276C mutation alters the formation of this normal intramolecular disulfide bond. The mutation in patient case 2, which has been so far not yet described in tumor patients, lies within the extracellular juxtamembrane domain. Interestingly, juxtamembrane FGFR2 germline mutations at S372C and Y375C have been reported in individuals with Beare–Stevenson syndrome, which results in a broad range of abnormalities in skeletal and skin development^31^. Moreover, homologous mutations in the mouse cause constitutive FGFR2 activation and abnormal skin and skull differentiation in transgenic models. In addition, paralogous mutations in *FGFR3* (G370C, S371C, and Y373C) have been associated with developmental limb abnormalities in humans^31^, suggesting that mutations in the juxtamembrane domains of FGFR2 and FGFR3 result in a similar activation mechanism. Thus, we propose that both gain-of-cysteine mutations lead to a ligand-independent constitutive FGFR activation, possibly by the formation of intermolecular disulfide bonds or a covalent dimerization of mutant receptor molecules.

In conclusion, our results suggest that functional characterization of *FGFR2* mutations, beyond the well-known fusion genes, might identify additional iCCA tumors that are dependent on this pathway. With a steadily growing number of available drugs that inhibit FGFR signaling, *FGFR2* alterations should be further characterized by in silico investigations and/or activation of downstream targets to enable patients’ selection for this treatment option. However, which drug is ideally suited to treat which FGFR2 alteration is one of the challenging questions that have to be investigated further. Moreover, as cysteine residues can be efficiently targeted by reducing or electrophilic agents, our finding might have implications also for the development of drugs targeting specific *FGFR2* mutations.

## Methods

### Patients and MTB organization

Patients were informed by a specialist in clinical genetics before they provided written informed consent for the collection of tumor samples and NGS analysis. They were referred to the MTB at Tübingen University Hospital and these cases are part of a retrospective, open-label analysis, which was reviewed and approved by the local ethics committee (873/2020BO). The MTB consisted of an interdisciplinary team coordinated by the Tuebingen Center for Personalized Medicine and includes experts in clinical and translational oncology, pathology, bioinformatics, molecular biology, radiology, and human genetics, as described previously^16^. Best response monitoring was performed by radiological imaging in line with RECIST 1.1.

### Histopathology and IHC

Hematoxylin–eosin staining was performed on 2.5 µm sections cut from formalin-fixed, paraffin-embedded (FFPE) tissue blocks. All histological slides were reviewed by an experienced pathologist with expertise in liver cancer pathology. The following immunostains were performed: pAKT(Thr308) (polyclonal rabbit/1:200/Merck Millipore, Darmstadt, Germany), pP44/42 MAPK(Thr202/Tyr204) (rabbit monoclonal, clone 20G11/1:400/CellSignaling, Cambridge, UK), pP38 MAPK(Thr180/Tyr182) (rabbit monoclonal, clone D3F9/1:800/CellSignaling), pSTAT1(Ser727) (rabbit monoclonal, clone EPR3146/1:1500/Abcam, Cambridge, UK), pSTAT3(Tyr705) (rabbit monoclonal, clone D3A7/1:400/CellSignaling), FRS2 (ABIN2855603/polyclonal rabbit/1:250/antibodies-online, Aachen, Germany), and YAP1 (EP1674Y/monoclonal rabbit/1:400/Abcam, Cambridge, UKx). DNA and RNA extraction for NGS analysis was performed from FFPE tissue sections using the Maxwell RSC FFPE Plus DNA Purification Kit (Promega, Fitchburg, WI) and the Maxwell RSC RNA FFPE Kit (Promega), respectively.

### In silico investigations

All molecular modeling and visualization were carried out with Schrödinger Suite releases 2020-1 and 2020-3 (Schrödinger, LLC, New York, NY). The extracellular structure of FGFR2 (F276C) was modeled based on the PDB-structure 1E0O^32^ with the Prime module. The c.1107_1113delinsCTCG alteration location was based on the topology assignment at UniProtKB database, entry P21802 (FGFR2_HUMAN).

### Genetic tissue characterization

Tumors were characterized by NGS panel sequencing of full coding sequences of more than 700 genes as previously described^16^. Details of the analysis are shown in Supplementary Tables 1 (Case 1) and 2 (Case 2) and the investigated genes in Supplementary Table. 4.

### Transcriptome analysis

Two hundred nanograms of total RNA from FFPE tumor samples was transcribed into cDNA. The New England Biolabs NEBNext Ultra II Directional RNA Library Prep Kit was used for library preparation and the sample was sequenced as 2 × 100 bp paired-end reads on an Illumina system. The raw data were demultiplexed using Illumina bcl2fastq and converted into FASTQ files. The data were further processed using the in-house bioinformatics pipeline megSAP (https://github.com/imgag/megSAP). The normalized gene expression FPKM (fragments per kilobase per million) and TPM (transcripts per kilobase per million) were calculated using the Subreads package from the featureCounts program^33^, which is freely available online. Fusions were identified with the freely available open-source software tool STAR-Fusion^34^.

### Comparison to TCGA cohort

The RNA-seq raw read counts from publicly available iCCA cohort were taken from TCGA level 3 data including 29 patients. The sample ID from the TCGA cohort of each patient is shown in Supplementary Table. 3. Among them, four patients had *FGFR2* fusion and one patient had an *FGFR2* gene mutation. We used the online available package DESeq2^35^ for normalization (mean-of-ratios), regularized log (rlog) transformation of the data, and differential expression analysis. Principal component analysis (PCA) was used to calculate the using rlog data.

### Reporting Summary

Further information on research design is available in the Nature Research Reporting Summary linked to this article.

## Supplementary information

Supplementary Material

REPORTING SUMMARY

## Data Availability

The NGS-panel sequencing dataset generated during the current study is not publicly available as these are patient samples with potentially identifiable germline information and there is no patient consent for depositing this sequencing data in a public repository. However, the data are available from the corresponding author on reasonable request.
