# Supplementary material for: Targeting extracellular and juxtamembrane FGFR2 mutations in chemotherapy-refractory cholangiocarcinoma
Source: NPJ Precis Oncol. 2021 Sep 3;5:80. doi: 10.1038/s41698-021-00220-0 (PMC8417271; doi:10.1038/s41698-021-00220-0)
Supplement: Supplementary file 1 — Supplementary Material [file 41698_2021_220_MOESM1_ESM.pdf]

a.

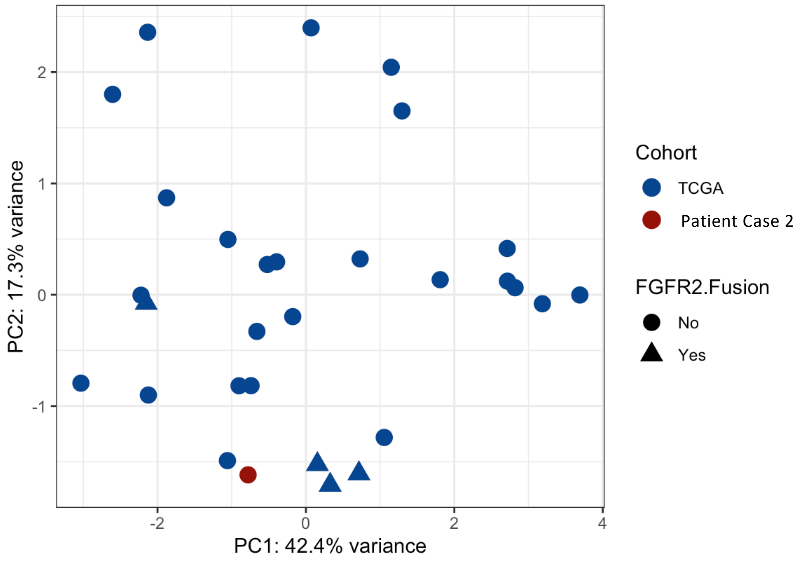

b.

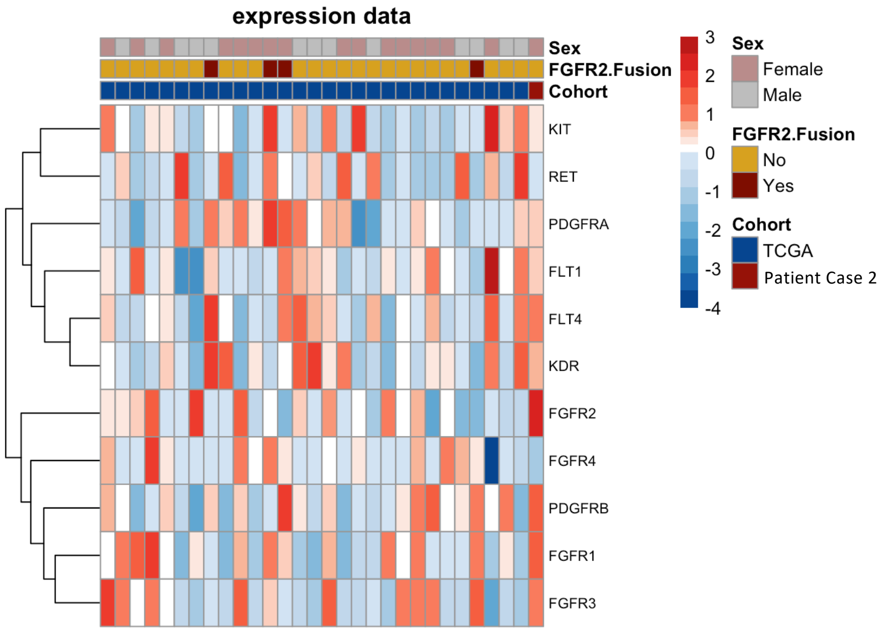

gene expression values are scaled per row (i,e, gene)

c.

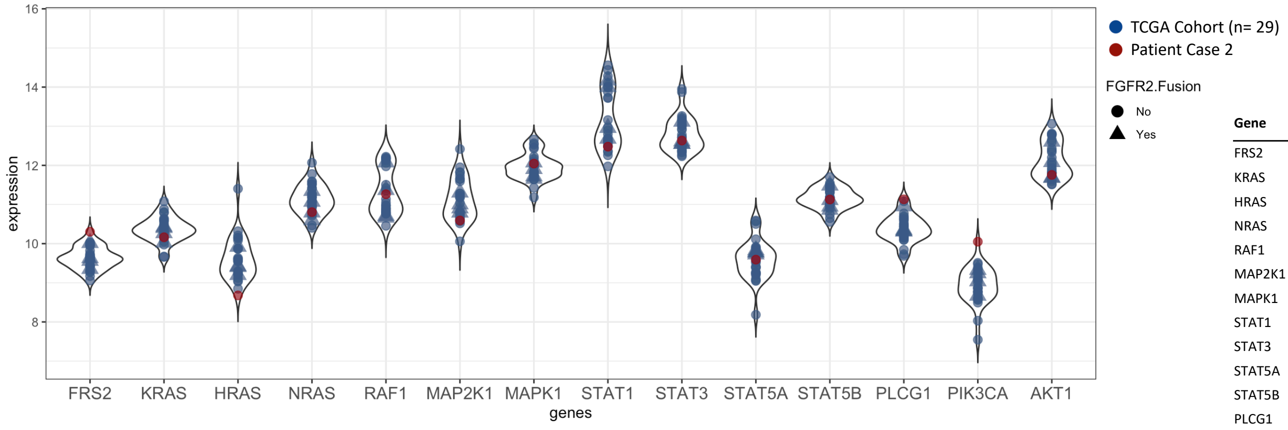

Gene	log2 fold change
FRS2	0,90
KRAS	-0,35
HRAS	-1,57
NRAS	-0,55
RAF1	-0,29
MAP2K1	-0,92
MAPK1	0,05
STAT1	-1,33
STAT3	-0,35
STAT5A	-0,15
STAT5B	-0,08
PLCG1	0,98
PIK3CA	1,46
AKT1	-0,65

Supplementary Figure 1

Supplementary Figure 1

Transcriptome analysis of tumor tissue sample from patient Case 2 in comparison with a cohort of 29 patients with cholangiocarcinoma from the TCGA cohort.

(TCGA Research Network, https://www.cancer.gov/tcga, data retrieved October 29th, 2020).

a PCA plot of patient Case 2 (red) in comparison to the TCGA cohort (blue). Samples with *FGFR2* fusion genes are shown as a triangle.

c Heatmap of the tyrosine kinases FGFR1-4, FLT1, FLT4, KDR, KIT, PDGFRA, PDGFRB, RET from the patient´s transcriptome data and of 29 patients with cholangiocarcinoma from the TCGA database. Results are shown with the highest expression in dark red (+3) and step-wise to the lowest expression in dark blue (-4).

d Transcriptome analysis with expression levels of selected FGFR2 downstream targets. Expression levels of FRS2, KRAS, NRAS, RAF1, MAP2K1, MAPK1, STAT1, STAT3, STAT5A, STAT5B, PLCG1 and AKT1 from the patient´s transcriptome data (red) and of 29 patients with cholangiocarcinoma from the TCGA database (blue). Samples with *FGFR2* fusion genes are shown as a triangle. The table shows the log2-fold change of the patient´s expression level in comparison to the TCGA cohort.

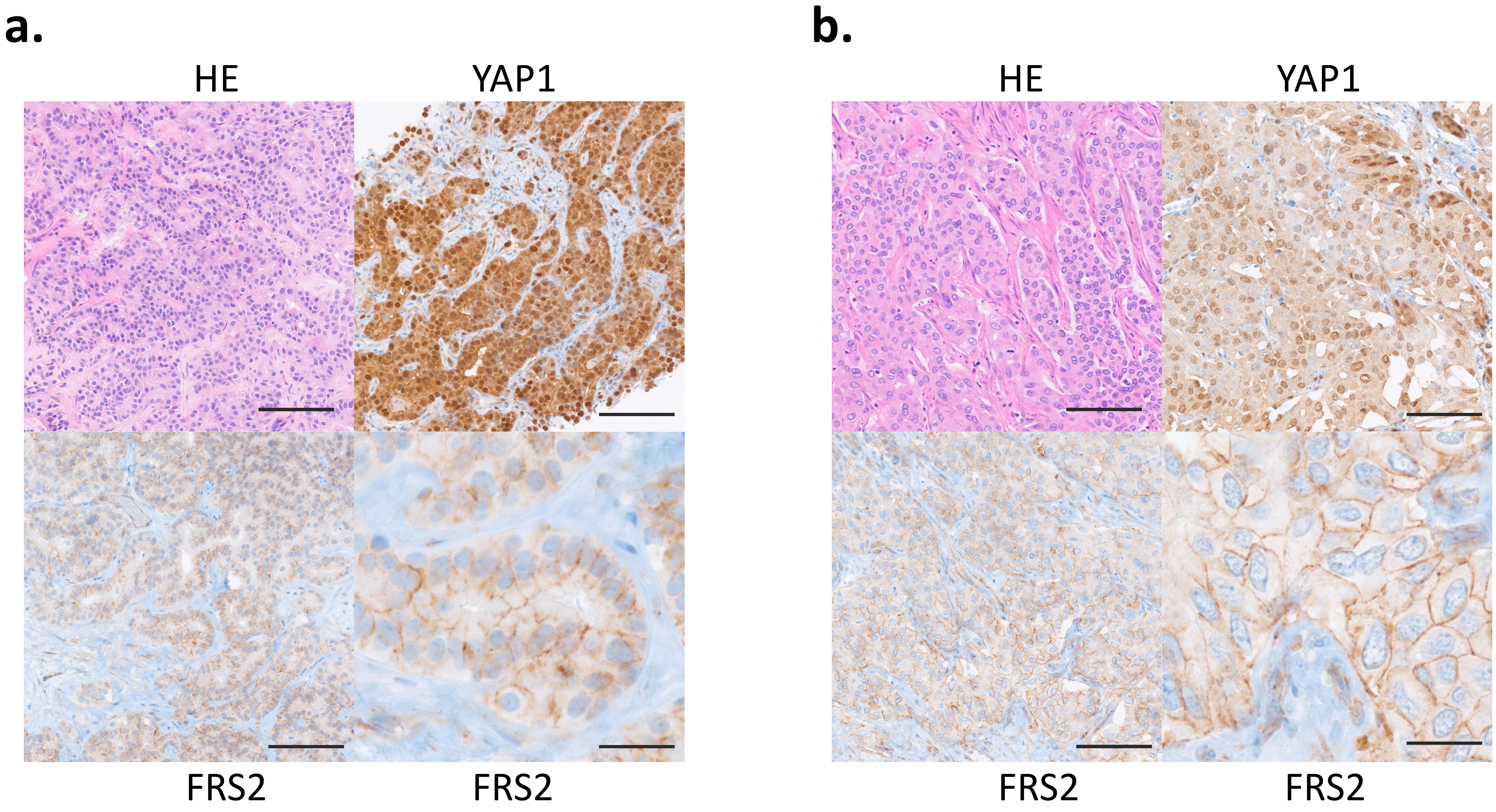

Scale bar 100µm, except lower right panel in each part 25µm

Supplementary Figure 2

Supplementary Figure 2

Immunohistochemistry of FGFR2 downstream targets for Case 1 (a) and Case 2 (b) at first diagnosis in April 2018.

Hematoxylin and Eosin staining (HE, upper part left), Yes-associated protein 1 (YAP1, upper part right), Fibroblast growth factor receptor substrate 2 (FRS2, lower part). Scale bar = 100µm (except lower left panel in each part: scale bar = 25µm).

Supplementary Table 1

NGS Panel (710 genes); Tumor mutational burden: 3.1 Var/Mbp; Tumor fraction histology 80%.

No relevant germline alterations were detected. All variants listed below represent somatic mutations.

Gene	Alteration	Variant	Copy number	Novel allele frequency
IDH1	SNV	p.Arg132Leu; NM_001282386.1	-	0.27
CSMD1	SNV	p.Trp1348Cys; NM_033225.5	-	0.31
FES	SNV	p.Ser412Leu; NM_002005.3	-	0.10
FGFR2	**SNV**	**p.Phe276Cys; NM_000141.4**	**-**	**0.49**
KMT2B	SNV	p.Arg1003Trp; NM_014727.2	-	0.24
SAV1	INDEL	p.Tyr357Leu*fs**38; NM_021818.3	-	0.11
CKS1B	CNV_focal	amplification	4	-
MDM4	CNV_non focal	amplification	4	-
MUC1	CNV_focal	amplification	4	-
VHL	CNV_non focal	heterozygous deletion	1	-
PIK3CA	CNV_non focal	Duplication	3	-
PIK3R1	CNV_non focal	heterozygous deletion	1	-
PTEN	CNV_non focal	heterozygous deletion	1	-
CCNE1	CNV_non focal	duplication	3	-
NOTCH3	CNV_non focal	duplication	3	-

SNV	single nucleotide variant
INDEL	insertion or deletion affecting 2 or more nucleotides
CNV_focal	copy number variation affecting listed gene only
CNV_non focal	copy number variation affecting a chromosomal region encompassing given gene [Genes within this region which are not considered disease relevant are not listed]

Supplementary Table 2

NGS Panel (708 genes); Tumor mutational burden: 4.5 Var/Mbp; Tumor fraction histology 50%.

No relevanmt germline alterations were detected. All variants listed below represent somatic mutations.

Gene	Alteration	Variant	Copy number	Novel allele frequency
ALK	SNV	p.Arg186Lys<br>ENST00000389048	-	0.09
	CNV_non focal	heterozygous deletion	1	-
CDK8	SNV	p.Thr196Ala<br>ENST00000381527	-	0.21
	CNV_cluster	heterozygous deletion	1	-
FANCM	SNV	p.Arg398Gln<br>ENST00000267430	-	0.11
FGFR2	**INDEL**	**p.Thr370_Ala371delinsCys**<br>ENST00000358487	**-**	**0.36**
	CNV_non focal	heterozygous deletion	1	-
FOXP1	SNV	p.Ala532Glu<br>ENST00000318789	-	0.37
	CNV_non focal	heterozygous deletion	1	-
KDM5C	SNV	p.Val507Gly<br>ENST00000375401	-	0.25
LATS2	INDEL	p.Asn654Ile*fs*Ter5<br>ENST00000382592	-	0.38
	CNV_cluster	heterozygous deletion	1	-
RASA1	INDEL	p.Val544His*fs*Ter29<br>ENST00000274376	-	0.15
	CNV_non focal	heterozygous deletion	1	-
ZNRF3	SNV	p.Gly655Glu<br>ENST00000544604	-	0.08
APC	CNV_non focal	heterozygous deletion	1	-
ATM	CNV_non focal	heterozygous deletion	1	-
AXIN1	CNV_non focal	heterozygous deletion	1	-
BAP1	CNV_non focal	heterozygous deletion	1	-
BIRC3	CNV_non focal	heterozygous deletion	1	-
BRCA2	CNV_non focal	heterozygous deletion	1	-
CDKN1A	CNV_non focal	heterozygous deletion	1	-
CDKN2A	CNV_non focal	heterozygous deletion	1	-
CDKN2C	CNV_non focal	heterozygous deletion	1	-
FANCC	CNV_non focal	heterozygous deletion	1	-
FANCD2	CNV_non focal	heterozygous deletion	1	-
FBXW7	CNV_non focal	heterozygous deletion	1	-
FLCN	CNV_non focal	heterozygous deletion	1	-
INPP4B	CNV_non focal	heterozygous deletion	1	-
MAP2K4	CNV_non focal	heterozygous deletion	1	-
MEN1	CNV_non focal	heterozygous deletion	1	-
MLH1	CNV_non focal	heterozygous deletion	1	-
MSH2	CNV_non focal	heterozygous deletion	1	-
PALB2	CNV_non focal	heterozygous deletion	1	-
PBRM1	CNV_non focal	heterozygous deletion	1	-
PRDM1	CNV_non focal	heterozygous deletion	1	-
PTEN	CNV_non focal	heterozygous deletion	1	-
RB1	CNV_non focal	heterozygous deletion	1	-

SETD2	CNV_non focal	heterozygous deletion	1	-
SMAD4	CNV_non focal	heterozygous deletion	1	-
SUFU	CNV_non focal	heterozygous deletion	1	-
TNFAIP3	CNV_non focal	heterozygous deletion	1	-
TP53	CNV_non focal	heterozygous deletion	1	-
TSC1	CNV_non focal	heterozygous deletion	1	-
TSC2	CNV_non focal	heterozygous deletion	1	-
VHL	CNV_non focal	heterozygous deletion	1	-
WT1	CNV_non focal	heterozygous deletion	1	-

SNV	single nucleotide variant
INDEL	insertion or deletion affecting 2 or more nucleotides
CNV_focal	copy number variation affecting listed gene only
CNV_cluster	copy number variation affecting listed genes and a region shorter than 25% of the chromosome
CNV_non focal	copy number variation affecting a chromosomal region encompassing given gene
	[Genes within these regions which are not considered disease relevant are not listed]

Patient ID	Sample ID	Cancer Type	Cancer Type Detailed	FGFR2 Fusion	Mutation Count	Fraction Genome Altered	Diagnosis Age	Sex	Ethnicity Category	Race Category	Tumor Site
TCGA-3X-AAVA	TCGA-3X-AAVA-01	Hepatobiliary Cancer	Intrahepatic Cholangiocarcinoma		84	0.2021	50	Female	NOT HISPANIC OR LATINO	WHITE	Bile duct
TCGA-3X-AAVE	TCGA-3X-AAVE-01	Hepatobiliary Cancer	Intrahepatic Cholangiocarcinoma		98	0.1498	60	Male	NOT HISPANIC OR LATINO	ASIAN	Bile duct
TCGA-4G-AAZO	TCGA-4G-AAZO-01	Hepatobiliary Cancer	Intrahepatic Cholangiocarcinoma		237	0.1333	71	Female	NOT HISPANIC OR LATINO	WHITE	Bile duct
TCGA-4G-AAZT	TCGA-4G-AAZT-01	Hepatobiliary Cancer	Intrahepatic Cholangiocarcinoma		101	0.2069	62	Male	NOT HISPANIC OR LATINO	WHITE	Bile duct
TCGA-W5-AA2G	TCGA-W5-AA2G-01	Hepatobiliary Cancer	Intrahepatic Cholangiocarcinoma		124	0.3791	62	Female	NOT HISPANIC OR LATINO	WHITE	Bile duct
TCGA-W5-AA2I	TCGA-W5-AA2I-01	Hepatobiliary Cancer	Intrahepatic Cholangiocarcinoma		106	0.4619	66	Male	NOT HISPANIC OR LATINO	WHITE	Bile duct
TCGA-W5-AA2O	TCGA-W5-AA2O-01	Hepatobiliary Cancer	Intrahepatic Cholangiocarcinoma		111	0.4669	57	Male	NOT HISPANIC OR LATINO	WHITE	Bile duct
TCGA-W5-AA2Q	TCGA-W5-AA2Q-01	Hepatobiliary Cancer	Intrahepatic Cholangiocarcinoma	FGFR2_KIAA1598	128	0.1980	68	Male	NOT HISPANIC OR LATINO	WHITE	Bile duct
TCGA-W5-AA2R	TCGA-W5-AA2R-01	Hepatobiliary Cancer	Intrahepatic Cholangiocarcinoma		102	0.2625	77	Female	NOT HISPANIC OR LATINO	WHITE	Bile duct
TCGA-W5-AA2T	TCGA-W5-AA2T-01	Hepatobiliary Cancer	Intrahepatic Cholangiocarcinoma		83	0.7373	64	Female	NOT HISPANIC OR LATINO	WHITE	Bile duct
TCGA-W5-AA2U	TCGA-W5-AA2U-01	Hepatobiliary Cancer	Intrahepatic Cholangiocarcinoma		152	0.3428	78	Female	NOT HISPANIC OR LATINO	WHITE	Bile duct
TCGA-W5-AA2W	TCGA-W5-AA2W-01	Hepatobiliary Cancer	Intrahepatic Cholangiocarcinoma	BICC1_FGFR2	87	0.2554	31	Female	NOT HISPANIC OR LATINO	WHITE	Bile duct
TCGA-W5-AA2Z	TCGA-W5-AA2Z-01	Hepatobiliary Cancer	Intrahepatic Cholangiocarcinoma	FGFR2_C10orf118	79	0.4081	29	Female	NOT HISPANIC OR LATINO	WHITE	Bile duct
TCGA-W5-AA30	TCGA-W5-AA30-01	Hepatobiliary Cancer	Intrahepatic Cholangiocarcinoma		128	0.4012	82	Male	NOT HISPANIC OR LATINO	WHITE	Bile duct
TCGA-W5-AA31	TCGA-W5-AA31-01	Hepatobiliary Cancer	Intrahepatic Cholangiocarcinoma		110	0.6386	71	Male	NOT HISPANIC OR LATINO	WHITE	Bile duct
TCGA-W5-AA33	TCGA-W5-AA33-01	Hepatobiliary Cancer	Intrahepatic Cholangiocarcinoma		71	0.2274	60	Male	NOT HISPANIC OR LATINO	WHITE	Bile duct
TCGA-W5-AA34	TCGA-W5-AA34-01	Hepatobiliary Cancer	Intrahepatic Cholangiocarcinoma		117	0.4024	75	Female	NOT HISPANIC OR LATINO	WHITE	Bile duct
TCGA-W5-AA38	TCGA-W5-AA38-01	Hepatobiliary Cancer	Intrahepatic Cholangiocarcinoma		109	0.2476	55	Female	NOT HISPANIC OR LATINO	WHITE	Bile duct
TCGA-W5-AA39	TCGA-W5-AA39-01	Hepatobiliary Cancer	Intrahepatic Cholangiocarcinoma		747	0.3159	81	Male	NOT HISPANIC OR LATINO	WHITE	Bile duct
TCGA-W6-AA0S	TCGA-W6-AA0S-01	Hepatobiliary Cancer	Intrahepatic Cholangiocarcinoma		100	0.2993	46	Female	HISPANIC OR LATINO	WHITE	Bile duct
TCGA-WD-A7RX	TCGA-WD-A7RX-01	Hepatobiliary Cancer	Intrahepatic Cholangiocarcinoma		111	0.3243	71	Female	HISPANIC OR LATINO	WHITE	Bile duct
TCGA-ZD-A8I3	TCGA-ZD-A8I3-01	Hepatobiliary Cancer	Intrahepatic Cholangiocarcinoma		102	0.6296	73	Female	NOT HISPANIC OR LATINO	ASIAN	Bile duct
TCGA-ZH-A8Y1	TCGA-ZH-A8Y1-01	Hepatobiliary Cancer	Intrahepatic Cholangiocarcinoma		80	0.2631	74	Female	NOT HISPANIC OR LATINO	WHITE	Bile duct
TCGA-ZH-A8Y2	TCGA-ZH-A8Y2-01	Hepatobiliary Cancer	Intrahepatic Cholangiocarcinoma		121	0.7470	59	Female	NOT HISPANIC OR LATINO	BLACK OR AFRICAN AMERICAN	Bile duct
TCGA-ZH-A8Y4	TCGA-ZH-A8Y4-01	Hepatobiliary Cancer	Intrahepatic Cholangiocarcinoma		129	0.5614	58	Male	NOT HISPANIC OR LATINO	WHITE	Bile duct
TCGA-ZH-A8Y5	TCGA-ZH-A8Y5-01	Hepatobiliary Cancer	Intrahepatic Cholangiocarcinoma	FGFR2_BICC1	82	0.3080	69	Male	NOT HISPANIC OR LATINO	WHITE	Bile duct
TCGA-ZH-A8Y6	TCGA-ZH-A8Y6-01	Hepatobiliary Cancer	Intrahepatic Cholangiocarcinoma		136	0.0012	41	Female	NOT HISPANIC OR LATINO	BLACK OR AFRICAN AMERICAN	Bile duct
TCGA-ZH-A8Y8	TCGA-ZH-A8Y8-01	Hepatobiliary Cancer	Intrahepatic Cholangiocarcinoma		115	0.4276	73	Male	NOT HISPANIC OR LATINO	WHITE	Bile duct
TCGA-ZU-A8S4	TCGA-ZU-A8S4-01	Hepatobiliary Cancer	Intrahepatic Cholangiocarcinoma		106	0.0026	52	Male	NOT HISPANIC OR LATINO	WHITE	Bile duct

TCGA cohort with transcriptome analysis and iCCA, retrieved October 29th, 2020

Supplementary Table 3

Supplementary Table 4a

Case 1, NGS Panel (711 genes)

ABL1	ABL2	ACD	AIP	AJUBA	AKT1	AKT2	AKT3	ALK	AMER1	ANKRD26
APC	AR	ARAF	ARHGAP35	ARID1A	ARID1B	ARID2	ARID5B	ASXL1	ASXL2	ATG2B
ATM	ATP1A1	ATR	ATRX	AURKA	AURKB	AURKC	AXIN1	AXIN2	AXL	B2M
BAP1	BARD1	BCL10	BCL11A	BCL11B	BCL2	BCL3	BCL6	BCL9	BCOR	BCORL1
BCR	BIRC2	BIRC3	BIRC5	BLM	BMPR1A	BRAF	BRCA1	BRCA2	BRD3	BRD4
BRIP1	BTK	BTNL2	BUB1B	C11ORF30	CALR	CAMK2G	CARD11	CASP8	CBFB	CBL
CBLB	CBLC	CCDC6	CCND1	CCND2	CCND3	CCNE1	CD274	CD38	CD52	CD58
CD79A	CD79B	CD82	CDC73	CDH1	CDH11	CDH2	CDK12	CDK4	CDK6	CDK8
CDKN1A	CDKN1B	CDKN1C	CDKN2A	CDKN2B	CDKN2C	CEBPA	CEP57	CHD1	CHD2	CHD4
CHEK1	CHEK2	CIC	CIITA	CKS1B	CNOT3	COL1A1	COMMD1	CREB1	CREBBP	CRKL
CRTC1	CRTC2	CSF1R	CSF2	CSF3R	CSMD1	CSNK1A1	CTCF	CTLA4	CTNNA1	CTNNB1
CUL4B	CUX1	CXCR4	CYLD	CYP2A7	DAXX	DCC	DDB2	DDR1	DDR2	DDX11
DDX3X	DDX41	DEK	DHFR	DICER1	DIS3	DIS3L2	DKC1	DNMT1	DNMT3A	DOT1L
DPYD	EBP	EGFR	EGLN1	EGR2	EGR3	ELAC2	ELANE	ELF3	EML4	EP300
EPAS1	EPCAM	EPHA2	EPHA3	EPHA4	EPHB4	EPHB6	ERBB2	ERBB3	ERBB4	ERCC1
ERCC2	ERCC3	ERCC4	ERCC5	ERG	ERRFI1	ESR1	ESR2	ETNK1	ETS1	ETV1
ETV4	ETV5	ETV6	EWSR1	EXO1	EXT1	EXT2	EZH1	EZH2	FAM175A	FAM46C
FAN1	FANCA	FANCB	FANCC	FANCD2	FANCE	FANCF	FANCG	FANCI	FANCL	FANCM
FAS	FAT1	FBXW7	FES	FGF10	FGF14	FGF19	FGF2	FGF23	FGF3	FGF4
FGF5	FGF6	FGFBP1	FGFR1	FGFR2	FGFR3	FGFR4	FH	FKBP1A	FLCN	FLI1
FLT1	FLT3	FLT4	FOXA1	FOXA2	FOXE1	FOXL2	FOXO1	FOXO3	FOXP1	FOXQ1
FRK	FRS2	FUBP1	FUS	FYN	G6PD	GABRA6	GALNT12	GATA1	GATA2	GATA3
GATA4	GATA6	GLDN	GLI1	GLI2	GNA11	GNA13	GNAQ	GNAS	GPC3	GPER1
GPR124	GREM1	GRIN2A	GRM3	GSK3A	H3F3A	HCK	HGF	HIF1A	HIST1H3B	HLA-A
HLA-B	HLA-C	HLA-DPA1	HLA-DPB1	HLA-DQA1	HLA-DQB1	HLA-DRA	HLA-DRB1	HLF	HMGA2	HMGN1
HMOX2	HNF1A	HNF1B	HOXB13	HOXD8	HRAS	HSD3B1	HSP90AA1	HSP90AB1	ID3	IDH1
IDH2	IFNGR1	IFNGR2	IGF1R	IGF2	IGF2R	IKBKB	IKBKE	IKZF1	IKZF3	IL1B
IL1RN	IL2	IL21R	IL6	IL6ST	IL7R	ING4	INPP4B	INPPL1	IRF1	IRS2
ITK	JAK1	JAK2	JAK3	JUN	KAT6A	KDM5A	KDM5C	KDM6A	KDR	KEAP1
KIAA1549	KIT	KLF2	KLF4	KLHDC8B	KLHL6	KMT2A	KMT2B	KMT2C	KMT2D	KRAS
LATS1	LATS2	LCK	LIG4	LIMK2	LMO1	LRP1B	LRRK2	LTK	LYN	LZTR1

MAD2L2	MAFB	MAGEA1	MAGI1	MAGI2	MAML1	MAP2K1	MAP2K2	MAP2K3	MAP2K4	MAP2K5
MAP2K6	MAP2K7	MAP3K1	MAP3K14	MAP3K3	MAP3K4	MAP3K6	MAPK1	MAPK11	MAPK12	MAPK3
MAPK8IP1	MAX	MBD1	MC1R	MCL1	MDC1	MDM2	MDM4	MECOM	MED12	MEF2B
MEN1	MET	MGA	MGMT	MITF	MLH1	MLH3	MLLT10	MLLT3	MN1	MPL
MRE11A	MS4A1	MSH2	MSH3	MSH4	MSH5	MSH6	MSR1	MST1R	MTHFR	MTOR
MTRR	MUC1	MUC16	MUTYH	MXI1	MYB	MYC	MYCL	MYCN	MYD88	MYH11
MYH9	NBN	NCOA1	NCOA3	NCOR1	NF1	NF2	NFE2L2	NFKB1	NFKB2	NFKBIA
NFKBIE	NIN	NLRC5	NOP10	NOTCH1	NOTCH2	NOTCH3	NOTCH4	NPM1	NQO1	NR1I3
NRAS	NRG2	NSD1	NT5C2	NTHL1	NTRK1	NTRK2	NTRK3	NUMA1	NUP98	PAK3
PALB2	PALLD	PARK2	PARP1	PARP2	PARP4	PAX3	PAX5	PAX7	PBK	PBRM1
PBX1	PDCD1	PDCD1LG2	PDF	PDGFA	PDGFB	PDGFC	PDGFD	PDGFRA	PDGFRB	PDK1
PGR	PHF6	PHOX2B	PIAS4	PIGA	PIK3C2A	PIK3C2B	PIK3C2G	PIK3CA	PIK3CB	PIK3CD
PIK3CG	PIK3R1	PIK3R2	PIK3R3	PIM1	PKHD1	PLCG1	PLCG2	PML	PMS1	PMS2
POLD1	POLE	POLH	POLQ	POT1	PPM1D	PRDM1	PRDM16	PREX2	PRF1	PRKAR1A
PRKCA	PRKD1	PRKDC	PROM2	PRSS1	PRX	PSIP1	PSMB1	PSMB10	PSMB2	PSMB5
PSMB8	PSMB9	PSMC3IP	PSPH	PTCH1	PTCH2	PTEN	PTGS2	PTK2	PTK7	PTPN11
PTPRC	PTPRD	PTPRT	RAC1	RAC2	RAD21	RAD50	RAD51	RAD51B	RAD51C	RAD51D
RAD54B	RAD54L	RAF1	RALGDS	RARA	RARB	RARG	RASA1	RASAL1	RB1	RBM10
RECQL4	REL	RET	RFC2	RFX5	RHBDF2	RHEB	RHOA	RICTOR	RINT1	RIPK1
RIT1	RNASEL	RNF2	RNF43	ROS1	RPL22	RPS20	RPS6KB1	RPTOR	RSF1	RUNX1
RYR1	SACS	SAMHD1	SAV1	SBDS	SCG5	SDHA	SDHAF2	SDHB	SDHC	SDHD
SEC23B	SEMA4A	SETBP1	SETD2	SETDB1	SF3B1	SGK1	SH2B1	SH2B3	SH2D1A	SHFM1
SHH	SIK2	SIN3A	SIRT1	SKP2	SLC26A3	SLIT2	SLX4	SMAD3	SMAD4	SMARCA4
SMARCB1	SMARCE1	SMC1A	SMC3	SMO	SOCS1	SOX11	SOX2	SOX9	SPEN	SPINK1
SPOP	SPRED1	SPTA1	SRC	SRD5A2	SRGAP1	SRP72	SRSF2	SSTR1	SSTR2	SSTR3
SSTR5	SSX1	STAG1	STAG2	STAT1	STAT3	STAT5A	STAT5B	STK11	SUFU	SUZ12
SYK	TAF1	TAF15	TAP1	TAP2	TBK1	TBL1XR1	TBX3	TCF3	TCF7L2	TCL1A
TEK	TERC	TERF2IP	TERT	TET1	TET2	TFE3	TGFBR2	TLR4	TLX1	TMEM127
TNF	TNFAIP3	TNFRSF11A	TNFRSF13B	TNFRSF14	TNFRSF1A	TNFRSF1B	TNFRSF25	TNFRSF8	TNFSF11	TNK2
TOP1	TOP2A	TP53	TP53BP1	TPX2	TRAF2	TRAF3	TRAF5	TRAF6	TRAF7	TRRAP
TSC1	TSC2	TSHR	TUBA4A	TUBB	TYMS	U2AF1	UBE2T	UBR5	UGT2B15	UGT2B7
UIMC1	UNG	USP34	USP9X	VEGFA	VEGFB	VHL	VKORC1	WAS	WASF3	WHSC1
WISP3	WRN	WT1	XIAP	XPA	XPC	XPO1	XRCC1	XRCC2	XRCC3	XRCC5
XRCC6	YAP1	ZFHX3	ZHX3	ZNF217	ZNRF3	ZRSR2				

Supplementary Table 4b

Case 2, NGS Panel (708 genes)

ABCB1	ABCB11	ABL1	ABL2	ABRAXAS1	ACO1	ACTB	ACVR1	ACVR1B	ACVR2A	ADAM10
ADCY1	ADGRA2	AJUBA	AKAP9	AKT1	AKT2	AKT3	ALK	ALOX12B	AMER1	ANK3
APAF1	APC	AR	ARAF	AREG	ARFRP1	ARHGAP26	ARHGAP35	ARHGEF6	ARID1A	ARID1B
ARID2	ARID5B	ASXL1	ASXL2	ATM	ATP1A1	ATR	ATRX	AURKA	AURKB	AXIN1
AXIN2	AXL	B2M	BAP1	BARD1	BBC3	BCL2	BCL2L1	BCL2L11	BCL2L2	BCL6
BCLAF1	BCOR	BCORL1	BCR	BIRC3	BLM	BMPR1A	BRAF	BRCA1	BRCA2	BRD4
BRIP1	BTG1	BTK	BUB1B	CA9	CAD	CALR	CARD11	CARM1	CASP8	CBFB
CBL	CBLB	CBLC	CCAR1	CCN6	CCND1	CCND2	CCND3	CCNE1	CD274	CD276
CD69	CD79A	CD79B	CDC27	CDC73	CDH1	CDK12	CDK2	CDK4	CDK6	CDK8
CDKN1A	CDKN1B	CDKN1C	CDKN2A	CDKN2B	CDKN2C	CEBPA	CHD1	CHD2	CHD3	CHD4
CHD8	CHEK1	CHEK2	CIC	CLTC	CNOT1	CNTNAP1	COL1A1	COL3A1	COL7A1	COP1
CRBN	CREB1	CREBBP	CRKL	CRLF2	CRTC1	CRTC3	CSDE1	CSF1R	CSF3R	CTCF
CTLA4	CTNNA1	CTNNB1	CTNND1	CTR9	CTTN	CUL1	CUL3	CUL4A	CUL4B	CUX1
CYLD	CYP17A1	CYP2C8	DAXX	DCUN1D1	DDB2	DDR1	DDR2	DDX3X	DDX5	DHCR7
DICER1	DIS3	DIS3L2	DKC1	DNM2	DNMT1	DNMT3A	DNMT3B	DOCK8	DOT1L	DPYD
DROSHA	E2F3	ECT2L	EED	EEF1A1	EGFL7	EGFR	EGR3	EIF1AX	EIF4A2	ELANE
ELF3	EML4	EMSY	EP300	EPCAM	EPHA2	EPHA3	EPHA5	EPHA7	EPHB1	EPHB2
EPHB6	ERBB2	ERBB3	ERBB4	ERCC1	ERCC2	ERCC3	ERCC4	ERCC5	ERCC6	EREG
ERG	ERRFI1	ESR1	ESR2	ETNK1	ETV1	ETV4	ETV5	ETV6	EWSR1	EXT1
EXT2	EZH1	EZH2	FAH	FANCA	FANCC	FANCD2	FANCE	FANCF	FANCG	FANCI
FANCL	FANCM	FAS	FAT1	FAT3	FBXO11	FBXW7	FES	FGF10	FGF14	FGF19
FGF23	FGF3	FGF4	FGF6	FGFBP1	FGFR1	FGFR2	FGFR3	FGFR4	FH	FLCN
FLI1	FLT1	FLT3	FLT4	FMR1	FN1	FOXA1	FOXA2	FOXE1	FOXL2	FOXP1
FOXQ1	FRS2	FUBP1	G6PD	GABRA6	GATA1	GATA2	GATA3	GATA4	GATA6	GBA
GID4	GJB2	GLI1	GLI2	GLI3	GNA11	GNA13	GNAI1	GNAQ	GNAS	GOLGA5
GPC3	GPS2	GREM1	GRIN2A	GRM3	GSK3B	H3F3A	H3F3B	H3F3C	HCFC1	HDAC2
HFE	HGF	HIF1A	HIST1H1C	HIST1H2BD	HIST1H3A	HIST1H3B	HIST1H3C	HIST1H3D	HIST1H3E	HIST1H3G
HIST1H3H	HIST1H3I	HIST1H3J	HLA-A	HLA-B	HMBS	HNF1A	HOXD8	HRAS	HSD3B1	HSP90AA1
HSP90AB1	HSPA8	ICOSLG	IDH1	IDH2	IFNGR1	IGF1	IGF1R	IGF2	IGF2R	IKBKB
IKBKE	IKZF1	IL10	IL7R	ING1	INHBA	INPP4A	INPP4B	INPPL1	INSR	IRF2
IRF4	IRS1	IRS2	IRS4	ITK	JAK1	JAK2	JAK3	JMJD1C	JUN	KAT6A

KCNJ5	KDM5A	KDM5C	KDM6A	KDR	KEAP1	KEL	KIT	KLF4	KLHL6	KMT2A
KMT2B	KMT2C	KMT2D	KRAS	L2HGDH	LATS1	LATS2	LCP1	LIFR	LMNA	LMO1
LPP	LRP1B	LRRK2	LYN	LZTR1	MAD2L2	MAGI2	MAML2	MAP2K1	MAP2K2	MAP2K4
MAP2K7	MAP3K1	MAP3K13	MAP3K3	MAP3K4	MAP4K1	MAP4K3	MAPK1	MAPK8IP1	MAX	MBD1
MC1R	MCL1	MDC1	MDM2	MDM4	MECOM	MED12	MED17	MED23	MEF2B	MEN1
MET	MGA	MITF	MKI67	MLH1	MLH3	MLLT10	MLLT3	MMP2	MN1	MNDA
MPL	MRE11	MSH2	MSH3	MSH6	MTAP	MTHFR	MTOR	MUTYH	MYB	MYC
MYCL	MYCN	MYD88	MYH9	MYOD1	NBN	NCOA3	NCOR1	NCOR2	NEDD4L	NF1
NF2	NFE2L2	NFKB1	NFKB2	NFKBIA	NFKBIE	NHP2	NIN	NKX2-1	NKX3-1	NOP10
NOTCH1	NOTCH2	NOTCH3	NOTCH4	NPM1	NQO1	NR4A2	NRAS	NRG1	NSD1	NSD2
NSD3	NT5C2	NTHL1	NTN4	NTRK1	NTRK2	NTRK3	NUMA1	NUP93	NUP98	NUTM1
PABPC1	PAK1	PAK3	PAK5	PALB2	PARP1	PARP2	PARP4	PAX5	PAX8	PBRM1
PCBP1	PDCD1	PDCD1LG2	PDGFB	PDGFRA	PDGFRB	PDK1	PDPK1	PHF6	PHOX2B	PIK3C2B
PIK3C2G	PIK3C3	PIK3CA	PIK3CB	PIK3CD	PIK3CG	PIK3R1	PIK3R2	PIK3R3	PIM1	PIP5K1A
PLCG1	PLCG2	PLK2	PMAIP1	PML	PMS1	PMS2	PNRC1	POLD1	POLE	POLH
POLQ	POT1	PPM1D	PPP2R1A	PPP6C	PRDM1	PRDM9	PREX2	PRF1	PRKAR1A	PRKCH
PRKCI	PRKDC	PRKN	PRPF8	PRSS1	PRSS8	PRX	PSIP1	PTCH1	PTEN	PTGS2
PTPN11	PTPRB	PTPRC	PTPRD	PTPRS	PTPRT	QKI	RAC1	RAD21	RAD50	RAD51
RAD51B	RAD51C	RAD51D	RAD52	RAD54L	RAF1	RANBP2	RARA	RASA1	RB1	RBM10
RECQL	RECQL4	REL	RET	RHBDF2	RHEB	RHOA	RICTOR	RIT1	RNF43	ROS1
RPGR	RPL10	RPL22	RPL5	RPS6KA4	RPS6KB2	RPTOR	RUNX1	RUNX1T1	RUNX3	RYR1
SACS	SAV1	SBDS	SDHA	SDHAF2	SDHB	SDHC	SDHD	SERPINA1	SERPINB3	SETBP1
SETD2	SETDB1	SF3B1	SGK1	SH2B3	SH2D1A	SHOC2	SIN3A	SLC25A13	SLC26A3	SLC29A1
SLC44A4	SLIT2	SLX4	SMAD2	SMAD3	SMAD4	SMARCA1	SMARCA2	SMARCA4	SMARCB1	SMARCD1
SMARCE1	SMC1A	SMC3	SMO	SNCAIP	SOCS1	SOS1	SOX10	SOX17	SOX2	SOX9
SPEN	SPOP	SPRTN	SPTA1	SPTAN1	SRC	SRSF2	SRY	STAG2	STAT3	STAT4
STAT5B	STK11	SUFU	SUZ12	SYK	TAF1	TAP1	TBL1XR1	TBX3	TCF12	TCF3
TCF7L2	TENT5C	TERT	TET1	TET2	TFAP2A	TFDP1	TGFBR1	TGFBR2	TJP2	TLR4
TMEM127	TMPRSS2	TNF	TNFAIP3	TNFRSF14	TNPO1	TOM1	TOP1	TOP2A	TP53	TP53BP1
TP63	TPMT	TPX2	TRAF3	TRAF7	TRIM37	TRIO	TRRAP	TSC1	TSC2	TSHR
TUBB3	TXNIP	TYMS	U2AF1	UBE2T	UBR5	UGT1A1	UPF3B	UROD	USP9X	VEGFA
VHL	WAS	WASF3	WNK1	WRN	WT1	XIAP	XPA	XPC	XPO1	XRCC2
XRCC3	YAP1	ZBTB16	ZBTB2	ZFHX3	ZFP36L1	ZFP36L2	ZMYM2	ZMYM3	ZNF217	ZNF703
ZNF750	ZNF814	ZNRF3	ZRSR2							
