# Supplementary material for: Targeting extracellular and juxtamembrane FGFR2 mutations in chemotherapy-refractory cholangiocarcinoma
Source: NPJ Precis Oncol. 2021 Sep 3;5:80. doi: 10.1038/s41698-021-00220-0 (PMC8417271; doi:10.1038/s41698-021-00220-0)
Supplement: Supplementary file 2 — REPORTING SUMMARY [file 41698_2021_220_MOESM2_ESM.pdf]

nature research

Corresponding author(s): Michael Bitzer

Last updated by author(s): June 18th, 2021

Reporting Summary

Nature Research wishes to improve the reproducibility of the work that we publish. This form provides structure for consistency and transparency in reporting. For further information on Nature Research policies, see our Editorial Policies and the Editorial Policy Checklist.

Statistics

For all statistical analyses, confirm that the following items are present in the figure legend, table legend, main text, or Methods section.

n/a	Confirmed	
☐	☒	The exact sample size (n) for each experimental group/condition, given as a discrete number and unit of measurement
☐	☒	A statement on whether measurements were taken from distinct samples or whether the same sample was measured repeatedly
☒	☐	The statistical test(s) used AND whether they are one- or two-sided<br>*Only common tests should be described solely by name; describe more complex techniques in the Methods section.*
☒	☐	A description of all covariates tested
☒	☐	A description of any assumptions or corrections, such as tests of normality and adjustment for multiple comparisons
☒	☐	A full description of the statistical parameters including central tendency (e.g. means) or other basic estimates (e.g. regression coefficient) AND variation (e.g. standard deviation) or associated estimates of uncertainty (e.g. confidence intervals)
☒	☐	For null hypothesis testing, the test statistic (e.g. F, t, r) with confidence intervals, effect sizes, degrees of freedom and P value noted<br>*Give P values as exact values whenever suitable.*
☒	☐	For Bayesian analysis, information on the choice of priors and Markov chain Monte Carlo settings
☒	☐	For hierarchical and complex designs, identification of the appropriate level for tests and full reporting of outcomes
☒	☐	Estimates of effect sizes (e.g. Cohen's d, Pearson's r), indicating how they were calculated

Our web collection on statistics for biologists contains articles on many of the points above.

Software and code

Policy information about availability of computer code

Data collection	In silico modeling investigations. All molecular modeling and visualization were carried out with Schrödinger Suite releases 2020-1 and 2020-3 (Schrödinger, LLC, New York, NY). The extracellular structure of FGFR2 (F276C) was modelled based on the PDB-structure 1E0O32 with Prime module. The c.1107_1113delinsCTCG alteration location was based on the topology assignment at UniProtKB database, entry P21802 (FGFR2_HUMAN).
Data analysis	Transcriptome analysis.:The raw data were demultiplexed using Illumina bcl2fastq and converted into FASTQ files. The data was further processed using the in-house bioinformatics pipeline megSAP (https://github.com/imgag/megSAP). The normalized gene expression FPKM (fragments per kilobase per million) and TPM (transcripts per kilobase per million) were calculated using the Subreads package (reference 33). Fusions were identified with STAR-Fusion (reference 34).<br>Comparison to TCGA cohort (TCGA level 3 data): The analysis performed in R version 4.0.4 and the DESeq2 R package version 1.30.1. We used DESeq2 (reference 35) for normalization (mean-of- ratios), regularized log (rlog) transformation of the data and differential expression analysis. Principal component analysis (PCA) calculated using rlog data.

For manuscripts utilizing custom algorithms or software that are central to the research but not yet described in published literature, software must be made available to editors and reviewers. We strongly encourage code deposition in a community repository (e.g. GitHub). See the Nature Research guidelines for submitting code & software for further information.

Data

Policy information about availability of data

All manuscripts must include a data availability statement. This statement should provide the following information, where applicable:

- Accession codes, unique identifiers, or web links for publicly available datasets
- A list of figures that have associated raw data
- A description of any restrictions on data availability

The NGS-panel sequencing dataset generated during the current study is not publicly available as these are patient samples with potentially identifiable germline information and there is no patient consent for depositing this sequencing data in a public repository. However, the data are available from the corresponding author on reasonable request.

Field-specific reporting

Please select the one below that is the best fit for your research. If you are not sure, read the appropriate sections before making your selection.

☒ Life sciences ☐ Behavioural & social sciences ☐ Ecological, evolutionary & environmental sciences

For a reference copy of the document with all sections, see nature.com/documents/nr-reporting-summary-flat.pdf

Life sciences study design

All studies must disclose on these points even when the disclosure is negative.

Sample size	The study includes two individual case reports
Data exclusions	n/a
Replication	The responses of the applied drugs were verfied during repeated imaging procedures
Randomization	No randomization for the two reported case studies
Blinding	No blinding for the two reported case studies

Reporting for specific materials, systems and methods

We require information from authors about some types of materials, experimental systems and methods used in many studies. Here, indicate whether each material, system or method listed is relevant to your study. If you are not sure if a list item applies to your research, read the appropriate section before selecting a response.

Materials & experimental systems

n/a	Involved in the study	
☐	☒	Antibodies
☒	☐	Eukaryotic cell lines
☒	☐	Palaeontology and archaeology
☒	☐	Animals and other organisms
☐	☒	Human research participants
☐	☒	Clinical data
☒	☐	Dual use research of concern

Methods

n/a	Involved in the study	
☒	☐	ChIP-seq
☒	☐	Flow cytometry
☒	☐	MRI-based neuroimaging

Antibodies

Antibodies used	pAKT(Thr308) (polyclonal rabbit/1:200/Merck Millipore, Darmstadt, Germany), pP44/42 MAPK(Thr202/Tyr204) (rabbit monoclonal, clone 20G11/1:400/CellSignaling, Cambridge, UK), pP38 MAPK(Thr180/Tyr182) (rabbit monoclonal, clone D3F9/1:800/CellSignaling), pSTAT1(Ser727) (rabbit monoclonal, clone EPR3146/1:1500/Abcam, Cambridge, UK), pSTAT3(Tyr705) (rabbit monoclonal, clone D3A7/1:400/ CellSignaling), FRS2 (ABIN2855603/polyclonal rabbit/1:250/antibodies-online, Aachen, Germany), and YAP1 (EP1674Y/ monoclonal rabbit/1:400/Abcam, Cambridge, United Kingdom)
Validation	IHC stainings with the above mentioned antibodies were performed on an IHC Platform used in routine diagnostics according to quality assurance requirements. Controls involved cytoblocks from modified human cell lines either with active or inactive/baseline FGFR-signalling pathway or normal/tumor tissue specimens. Each antibody was optimized in terms of concentrations and pretreatment/antigen retrieval.

Human research participants

Policy information about studies involving human research participants

Population characteristics	Two individual case report studies are reported
Recruitment	Both patients were presented to the Molecular Tumor Board of Tuebingen University
Ethics oversight	Ethics Committee of the Medical Faculty, Tuebingen University, Germany (whic is mentioned within the manuscript)

Note that full information on the approval of the study protocol must also be provided in the manuscript.

Clinical data

Policy information about clinical studies

All manuscripts should comply with the ICMJE guidelines for publication of clinical research and a completed CONSORT checklist must be included with all submissions.

Clinical trial registration	No registration as the study includes two individual case reports
Study protocol	No study protocol as the study includes two individual case reports
Data collection	No study protocol as the study includes two individual case reports
Outcomes	No study protocolas the study includes two individual case reports
